# Scanner-efficient diffusion tensor imaging of human cardiac microstructure using the fast composite splitting reconstruction algorithm

**DOI:** 10.1186/1532-429X-16-S1-W5

**Published:** 2014-01-16

**Authors:** Archontis Giannakidis, Pedro Ferreira, Andrew D Scott, Sonia Nielles-Vallespin, Sonya V Babu-Narayan, Philip J Kilner, Dudley J Pennell, David Firmin

**Affiliations:** 1 Cardiovascular Biomedical Research Unit, Royal Brompton Hospital, London, UK; 2 National Heart and Lung Institute, Imperial College, London, UK; 3 National Heart Lung and Blood Institute, National Institutes of Health, Bethesda, Maryland, USA

## Background

Diffusion tensor imaging (DTI) has been shown [1] to be extremely promising for characterizing the hierarchical microstructure of myocardium. DTI studies are hampered by lengthy acquisition times, needed for high spatial resolution and/or improved SNR. Compressed sensing (CS) algorithms recover data from under-sampled acquisitions, and have been used [2] to reduce scan time in MRI. The fast composite splitting algorithm (FCSA) [3] impressively outperforms other classical CS reconstruction methods by providing more accurate results in less CPU time. In this study, we investigate the feasibility of applying FCSA CS to DTI of an excised human heart. To our knowledge, this is the first time CS reconstruction has been applied to DTI of a human heart.

## Methods

### MRI

3D DTI of a whole human heart was performed on a 3T Siemens Skyra using a monopolar spin echo sequence with 30 diffusion directions at *b *= 770 smm^-2 ^(+b0) with 1 mm isotropic resolution.

### CS

**Figure 1:**
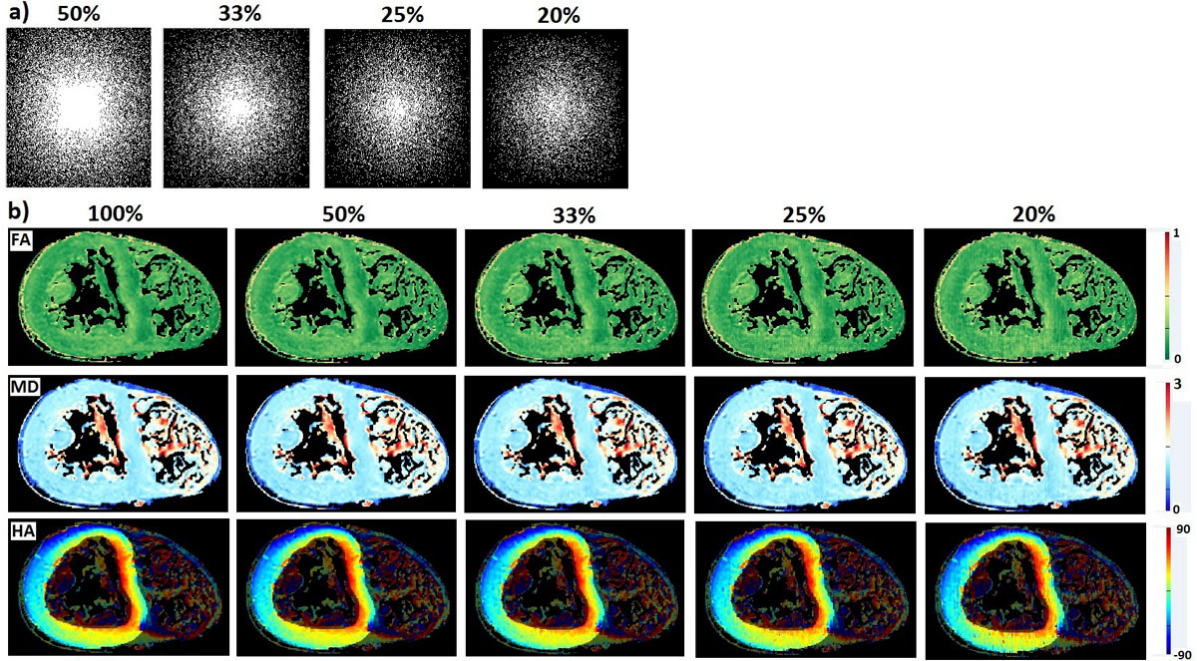


Efficient variable-density under-sampling of the *k*_y_-*k*_z _plane of the 3D Cartesian acquisition was applied retrospectively to the fully-sampled DTI dataset with sampling ratios 50, 33, 25, and 20% (sampling patterns are shown in Figure 1a). All under-sampled data were reconstructed with FCSA (*L*_1_-norm and total variation).

### Data analysis

Diffusion tensor data was reconstructed and the left-ventricular wall was analyzed. Maps of fractional anisotropy (FA), mean diffusivity (MD), and helix angles (HA) were computed for the fully-sampled and reconstructed under-sampled datasets. To evaluate the accuracy of FCSA CS, root mean square errors (RMSEs) of FA, MD, and HA were estimated between the full-sampled and the accelerated data-sets. All computations were performed using custom-made software in Matlab.

## Results

Figure 1b depicts maps of FA (1st row), MD (2nd row), HA (3rd row) for the fully, 50, 33, 25, and 20% sampled datasets. The quality of the maps produced by FCSA CS is comparable to the maps obtained from the fully-sampled data. Table 1 summarizes the RMSE for all DTI-derived parameters and acceleration factors. We find that even though the RSME values increase with the acceleration factor, the loss of information is minor. We conclude that essential information on cardiac diffusion properties is preserved up to an acceleration factor of 4. The strong convergence properties of FCSA were confirmed.

**Table 1:** Root-mean square error (RMSE) of fractional anisotropy (FA), mean diffusivity (MD), and helix angles (HA) for the 4 sampling ratios.

	FA	MD (10^-3 ^mm^2^/s)	HA
50.00%	0.0099	0.0166	3.3107

33.00%	0.0169	0.0296	5.4596

25.00%	0.0241	0.0390	7.1005

20.00%	0.0318	0.0496	9.2922

The mean FA value is 0.2961, and the mean MD value is 0.6993.

## Conclusions

We have demonstrated that CS using FCSA has potential to shorten acquisition times of cardiac DTI without compromising accuracy. These results can be used to minimize patient discomfort and mitigate growing healthcare costs through increasing contemporary scanner throughput.

## Funding

This work was supported by the National Institute of Health Research Cardiovascular Biomedical Research Unit at the Royal Brompton Hospital and Imperial College, London.

## References

[B1] Nielles-Valespin S MRM 2013

[B2] Lustig M MRM 2010

[B3] Huang J MedIA 2011

